# Dietary Diversity, Dietary Patterns, and Cardiometabolic Health in University Students: A Cross-Sectional Study

**DOI:** 10.3390/nu18030511

**Published:** 2026-02-02

**Authors:** Diana Fonseca-Pérez, Ludwig Álvarez-Córdova, Cecilia Arteaga-Pazmiño, Víctor Sierra-Nieto, Jaen Cagua-Ordoñez, Evelyn Frias-Toral, Giovanna Muscogiuri, Claudia Reytor-González, Daniel Simancas-Racines

**Affiliations:** 1 Carrera de Nutrición y Dietética, Facultad de Ciencias de la Salud, Universidad Católica de Santiago de Guayaquil, Guayaquil 090615, Ecuador; diana.fonseca@cu.ucsg.edu.ec; 2 Maestría de Nutrición y Dietética, Facultad de Ciencias de la Salud, Universidad de Las Américas (UDLA), Quito 170503, Ecuador; ludwig.alvarez@udla.edu.ec; 3 Carrera de Nutrición y Dietética, Facultad de Ciencias Médicas, Universidad de Guayaquil, Guayaquil 090615, Ecuador; cecilia.arteagap@ug.edu.ec; 4 Carrera de Fisioterapia, Facultad de Ciencias de la Salud, Universidad Católica de Santiago de Guayaquil, Guayaquil 090615, Ecuador; victor.sierra@cu.ucsg.edu.ec; 5 Facultad de Ciencias de la Salud y Bienestar Humano, Universidad Tecnológica Indoamérica, Quito 170103, Ecuador; jaencarlos_87@hotmail.com; 6 Escuela de Medicina, Universidad Espíritu Santo, Samborondón 0901952, Ecuador; evelynft@gmail.com; 7 Division of Research, Texas State University, 601 University Dr., San Marcos, TX 78666, USA; 8 Endocrinology Unit, Department of Clinical Medicine and Surgery, Federico II University, 80131 Naples, Italy; giovanna.muscogiuri@gmail.com; 9 Italian Centre for the Care and Wellbeing of Patients with Obesity, Federico II University Hospital, 80131 Naples, Italy; 10 UNESCO Chair on Health Education and Sustainable Development, Federico II University, 80131 Naples, Italy; 11 Facultad de Ciencias de la Salud y Bienestar Humano, Universidad Tecnológica Indoamérica, Ambato 180150, Ecuador; claudiareytor@gmail.com

**Keywords:** dietary diversity, dietary patterns, cardiometabolic risk, body composition, muscle mass, university students, Latin America, young adults

## Abstract

**Background:** Cardiometabolic risk is increasingly observed in young adults, particularly during university years, and is not limited to individuals with elevated body mass index. Emerging evidence highlights the presence of normal weight obesity—characterized by excess adiposity and unfavorable body composition despite normal BMI—which may confer early metabolic vulnerability. Dietary diversity is often promoted as a marker of dietary adequacy; however, its relationship with adiposity, body composition, and muscular health remains inconsistent, particularly in Latin American populations. Moreover, few studies have directly contrasted dietary diversity indicators with empirically derived dietary patterns in relation to cardiometabolic and functional outcomes. **Objective:** To examine the associations between dietary diversity, dietary patterns, and indicators of adiposity, muscular strength, and relative muscle mass in Ecuadorian university students. **Methods:** A cross-sectional study was conducted among 349 undergraduate students aged 18–26 years enrolled in health sciences programs in Ecuador. Dietary intake was assessed using a validated food frequency questionnaire. Dietary diversity was quantified using the Food and Agriculture Organization’s Individual Dietary Diversity Score, while dietary patterns were identified through principal component analysis followed by k-means clustering. Outcomes included excess body weight, relative muscle mass assessed by bioelectrical impedance analysis, and handgrip strength. Multivariable Poisson and linear regression models were fitted, adjusting for age, sex, academic program, physical activity level, and pre-existing conditions. **Results:** Despite their young age and low prevalence of diagnosed disease, approximately one-third of the participants exhibited markers of early cardiometabolic risk, including excess body weight and central adiposity. Higher dietary diversity was independently associated with a higher prevalence of excess body weight (adjusted prevalence ratio per one-unit increase in IDDS: 1.17; 95% CI: 1.06–1.30) and with greater relative muscle mass (adjusted β = 0.13; 95% CI: 0.05–0.22), whereas no association was observed with handgrip strength. In contrast, dietary patterns derived from multivariate analysis showed no significant associations with adiposity, muscular strength, or relative muscle mass after adjustment. **Conclusions:** In this young adult population, dietary diversity captured aspects of overall dietary exposure associated with both increased adiposity and greater lean mass, but not with muscular strength. Empirically derived dietary patterns demonstrated limited discriminatory capacity, likely reflecting dietary homogeneity within the cohort. These findings indicate that dietary diversity alone does not necessarily reflect diet quality and underscore the importance of interpreting diversity metrics alongside indicators of food quality, energy density, and body composition when evaluating early cardiometabolic risk in contemporary food environments.

## 1. Introduction

The global rise in overweight, obesity, and cardiometabolic risk is no longer confined to middle-aged or older populations, increasingly affecting young adults during critical life transitions such as university enrollment [1,2]. While this phenomenon is often framed using body mass index (BMI)- based definitions, cardiometabolic vulnerability in young adults is not limited to individuals with elevated BMI. A growing body of evidence describes the phenotype of normal-weight obesity, characterized by excess adiposity and unfavorable body composition despite a normal BMI [3,4]. This condition has been shown to be relatively prevalent among young adults and is associated with increased visceral fat accumulation, reduced lean mass, insulin resistance, and early cardiometabolic alterations, despite the absence of overt overweight or obesity classifications [3,5]. These findings underscore the limitations of BMI-based assessments and highlight the importance of incorporating body composition and fat distribution measures when evaluating early metabolic risk in young adults [6,7].

The transition to university life is characterized by substantial changes in dietary habits, physical activity patterns, sleep timing, and autonomy over food choices, all of which may collectively shape long-term metabolic trajectories [8,9,10]. Early adulthood is also marked by pronounced alterations in sleep timing and sleep duration, including late-night wakefulness, irregular sleep patterns, and circadian misalignment between eating behavior and endogenous rhythms [11,12]. These behaviors are common among individuals aged 18–30 years and have been linked to higher total energy intake, increased frequency of eating occasions, greater consumption of energy-dense foods, and less favorable anthropometric profiles [13]. The co-occurrence of irregular sleep, suboptimal dietary behaviors, and reduced structured physical activity may contribute to increases in fat mass and early cardiometabolic vulnerability, even in otherwise healthy young adults.

Dietary diversity has been widely promoted as a proxy indicator of dietary adequacy and micronutrient sufficiency, particularly in population-based assessments [14]. Conceptually, a more diverse diet increases the likelihood of consuming a broader range of foods and essential nutrients and has been associated with improved nutritional status across multiple settings [15,16]. However, dietary diversity does not inherently capture food quality, energy density, degree of processing, or the balance between nutrient-dense and ultra-processed foods [17,18,19]. In contemporary food environments, higher dietary diversity may therefore coexist with excessive caloric intake and unfavorable metabolic outcomes, particularly in urban and middle-income contexts where ultra-processed foods are widely available [14,20,21].

Consistent with this complexity, evidence linking dietary diversity to cardiometabolic health remains inconsistent, particularly among young adult populations [22,23]. While some studies report inverse associations between dietary diversity and adiposity or metabolic risk [22], others describe neutral or even positive associations with body weight, potentially reflecting greater overall food availability and consumption volume rather than healthier dietary composition [21,24]. These discrepancies highlight the need to interpret dietary diversity within the context of broader dietary patterns and co-occurring lifestyle behaviors, rather than as an isolated marker of diet quality.

Data-driven dietary pattern analyses, such as principal component analysis (PCA), provide complementary information by capturing habitual combinations of foods and their covariance structure within a population [25,26]. In contrast to diversity metrics, dietary patterns reflect how foods are consumed together rather than the number of food groups consumed. However, dietary pattern approaches may offer limited exposure contrast in relatively homogeneous populations, such as university students enrolled in health-related academic programs and sharing similar food environments [27]. Few studies have directly compared simple dietary diversity metrics with empirically derived dietary patterns while simultaneously evaluating their associations with body composition, muscular strength, and cardiometabolic indicators [28,29].

Additionally, muscular strength represents a relevant functional health marker in young adults, its determinants differ from those of adiposity and lean mass [30]. In this age group, muscle strength is strongly influenced by physical activity, sex-related physiological differences, and neuromuscular adaptation, whereas dietary factors may play a more modest or context-dependent role [31,32]. Importantly, higher estimates of skeletal muscle mass—particularly when assessed using bioelectrical impedance analysis—may partially reflect greater overall body mass or fat mass, rather than improved muscle quality or functional capacity [33]. Distinguishing between muscle quantity and muscle function is therefore essential when interpreting associations between diet and musculoskeletal health outcomes [30].

Young adult populations in Latin America remain underrepresented in nutritional epidemiology, despite rapid nutrition transitions, increasing consumption of ultra-processed foods, and rising prevalence of early metabolic risk [34,35,36,37,38]. Understanding how different operationalizations of diet relate to adiposity, body composition, and functional health in this context is essential for designing feasible, population-appropriate preventive strategies [39].

Therefore, the present study aimed to examine the associations between dietary diversity, dietary patterns, and cardiometabolic and functional health indicators in a cohort of university students in Ecuador. Specifically, we evaluated the relationships of the Individual Dietary Diversity Score (IDDS) and data-driven dietary patterns with excess body weight, relative muscle mass, and handgrip strength. By contrasting simple diversity metrics with multivariate dietary constructs, this study sought to clarify the extent to which dietary diversity reflects beneficial versus potentially adverse nutritional exposures in early adulthood.

## 2. Methods

### 2.1. Study Design

This observational, cross-sectional, and analytical study was conducted between November 2022 and February 2023 among undergraduate students enrolled in the Faculty of Health Sciences at the Universidad Católica de Santiago de Guayaquil (UCSG), Ecuador. The study was designed and reported in accordance with the Strengthening the Reporting of Observational Studies in Epidemiology (STROBE) guidelines [40]. Recruitment was carried out through an institutional online announcement, and all evaluations were conducted in person at the university’s Biomedicine Laboratory. The research protocol received ethical approval from the Ethics Committee for Research in Human Subjects of Hospital Clínica Kennedy, Guayaquil, Ecuador (CEISH No. HCK-CEISH-20-0001). All participants were informed about the study objectives, procedures, and confidentiality safeguards and provided written informed consent prior to enrollment.

### 2.2. Participants

Eligible participants were undergraduate students aged 18 to 26 years. The minimum required sample size was estimated using a finite population formula, assuming a 5% margin of error and a 95% confidence level. Exclusion criteria included pregnancy; the presence of implanted electronic devices or metallic prostheses; diagnosed eating disorders or chronic conditions known to affect dietary intake or body composition; acute illness on the day of evaluation; recent vigorous physical activity prior to assessment; or lack of informed consent. After applying these criteria and completing data quality control procedures, a total of 349 students met eligibility requirements and were included in the final analytical dataset. One participant was excluded during data cleaning due to incomplete dietary information. A recruitment flowchart summarizing participant selection and exclusions is provided to enhance transparency.

### 2.3. Sociodemographic Data

Sociodemographic information was collected using a structured questionnaire and included age, sex, nationality, academic program, and self-reported pre-existing health conditions. Academic programs comprised Medicine, Nutrition and Dietetics, Physiotherapy, and Odontology. Data completeness and internal consistency were verified prior to analysis to ensure accuracy and reliability.

### 2.4. Anthropometric and Body Composition Assessments

Anthropometric measurements were performed following standardized procedures established by the International Society for the Advancement of Kinanthropometry (ISAK). Body weight was measured using a calibrated digital scale, and height was measured with a fixed stadiometer. Waist and hip circumferences were obtained using a non-elastic measuring tape at standardized anatomical reference points. These measurements were used to calculate body mass index (BMI), waist-to-hip ratio (WHR), and waist-to-height ratio (WHtR). Body composition was assessed using multifrequency bioelectrical impedance analysis (BIA) with the SECA mBCA 525 device (seca GmbH & Co. KG, Hamburg, Germany). All assessments were conducted under standardized pre-test conditions, including adequate hydration, avoidance of vigorous physical activity prior to evaluation, and removal of metallic accessories. The BIA outputs included fat mass, fat-free mass, percent body fat, muscle mass, and visceral fat rating. It is important to note that BIA-derived muscle mass estimates reflect muscle quantity rather than muscle quality or functional capacity.

### 2.5. Cardiometabolic Risk Classification

Cardiometabolic risk indicators were defined using established and validated cut-off points. Abdominal obesity was classified according to International Diabetes Federation (IDF) criteria for Latin America (waist circumference ≥90 cm in men and ≥80 cm in women). Cardiovascular risk was defined using World Health Organization (WHO) waist-to-hip ratio thresholds (>0.90 for men and >0.85 for women). Metabolic risk was assessed using the waist-to-height ratio, applying the widely accepted cut-off of ≥0.50. Visceral adiposity was categorized based on device-specific reference standards as normal (values 1–12) or high/very high (values ≥ 13). These cardiometabolic indicators were used for descriptive purposes and were intentionally not included as covariates in regression models to avoid overadjustment for variables that may lie along the causal pathway between dietary exposures and body composition outcomes.

### 2.6. Dietary Assessment and IDDS

Dietary intake was assessed using a validated 91-item Food Frequency Questionnaire (FFQ) developed for the Ecuadorian population. Participants reported habitual consumption frequencies for each food item, which were converted into daily equivalents using standardized conversion algorithms.

Food items were subsequently mapped onto the ten food groups defined by the Individual Dietary Diversity Score (IDDS) proposed by the Food and Agriculture Organization of the United Nations (FAO): staples; pulses; nuts and seeds; dairy; flesh foods; eggs; dark green leafy vegetables; vitamin A–rich fruits and vegetables; other vegetables; and other fruits. Each food group was scored as “consumed” when the corresponding daily equivalent exceeded zero, and the total IDDS ranged from 0 to 10, with higher scores indicating greater dietary diversity.

IDDS was analyzed both as a continuous variable and categorized into tertiles representing low, medium, and high dietary diversity. This dual operationalization allowed evaluation of linear associations across the full distribution as well as potential threshold effects. As a qualitative indicator, IDDS captures the breadth of food group exposure but does not account for portion size, energy density, food processing level, or preparation methods; therefore, it was interpreted as a marker of dietary variety rather than dietary quality.

### 2.7. Construction of Dietary Frequency Domains and Dietary Patterns

To characterize broader habitual dietary behaviors beyond food group diversity, individual FFQ items were aggregated into seven quantitative dietary frequency domains: cereals and tubers; fruits; vegetables and legumes; animal-source proteins and dairy; fats and oils; sugars and sweets; and beverages, snacks, and ultra-processed products. For each participant, daily intake fractions were summed within each domain, generating continuous quantitative indicators of habitual consumption. Prior to pattern derivation, all domains were standardized to z-scores to ensure comparability.

Dietary patterns were derived using principal component analysis (PCA) with varimax rotation. PCA was selected as an exploratory, data-driven method to summarize prevailing dietary behaviors in the absence of predefined intermediate biomarkers, which are required for approaches such as reduced rank regression. Given the cross-sectional design and the relatively homogeneous dietary environment of university students enrolled in health-related academic programs, PCA was considered appropriate for capturing dominant covariance structures among food consumption domains.

Sampling adequacy was assessed using the Kaiser–Meyer–Olkin (KMO) measure, and Bartlett’s test of sphericity was used to confirm suitability for factor analysis. Components were retained based on eigenvalues greater than one, inspection of the scree plot, and nutritional interpretability. Standardized component scores were calculated for each retained component.

To enhance interpretability and facilitate classification of individuals into mutually exclusive dietary profiles, standardized PCA component scores were subsequently used as inputs for k-means clustering. This two-step approach allows translation of continuous dietary dimensions into discrete dietary patterns that are easier to describe and compare across individuals. The optimal number of clusters was determined using the elbow criterion, silhouette metrics, and conceptual coherence. The final solution identified three dietary patterns: a Low-Intake pattern, characterized by globally low consumption across domains; an Energy-Dense/Ultra-Processed pattern, reflecting higher intake of sugars, fats, snacks, and refined carbohydrates; and a Prudent/Whole-Food pattern, marked by greater intake of fruits, vegetables, legumes, and animal-source proteins.

We acknowledge that clustering procedures may reduce exposure contrast and statistical power, particularly in relatively homogeneous populations. Accordingly, PCA-derived dietary patterns were interpreted as descriptive summaries of habitual intake rather than as etiological or causal constructs.

### 2.8. Data Quality Control

A multilayer data quality control procedure was implemented prior to analysis. All variables were screened for physiological plausibility, and outliers were identified using Tukey’s method. Cross-variable consistency was examined across anthropometric, body composition, and dietary indicators to detect potential data entry or processing errors.

Distributions of continuous variables were visually inspected using histograms, boxplots, and Q–Q plots. The mapping of FFQ items to FAO-IDDS food groups was manually verified in a random subset of participants to ensure accuracy and consistency. Multicollinearity among covariates was assessed using the generalized variance inflation factor (GVIF), with all values below 5, indicating negligible collinearity and stable model estimation.

### 2.9. Statistical Analysis

All statistical analyses were conducted using RStudio version 4.5. Continuous variables were assessed for normality using histograms, Q–Q plots, and the Shapiro–Wilk test. Normally distributed variables were summarized as mean ± standard deviation, whereas skewed variables were described using median and interquartile range. Categorical variables were presented as absolute and relative frequencies. Group comparisons were performed using Student’s *t*-test or the Mann–Whitney U test for continuous variables, and the Chi-square test or Fisher’s exact test for categorical variables, as appropriate. Linear trends across IDDS tertiles were evaluated using the Cochran–Armitage trend test or analysis of variance with contrast testing, depending on variable type.

Bivariable associations between dietary exposures and health outcomes were initially examined. For dichotomous outcomes (excess body weight and low handgrip strength), Poisson regression models with robust variance estimators were fitted to obtain crude prevalence ratios (PRs) and 95% confidence intervals. For relative muscle mass, linear regression models with heteroscedasticity-robust standard errors were used.

Multivariable models were subsequently constructed using the same analytical frameworks. Covariates were selected a priori based on epidemiological relevance and biological plausibility and included age, sex, academic program, physical activity level, and self-reported pre-existing health conditions. Dietary exposures—IDDS (modeled as a continuous variable and as tertiles) and PCA-derived dietary patterns—were treated as primary exposure variables. Measures of central adiposity were intentionally excluded as covariates to avoid overadjustment for variables that may lie along the causal pathway between dietary exposures and body composition outcomes.

Adjusted marginal predicted values and their 95% confidence intervals were estimated using the margins package and visualized using ggplot2 in R software (version 4.5.1). Model assumptions were carefully evaluated. Multicollinearity was assessed using GVIF values, all of which were well below accepted thresholds. Overdispersion in Poisson models was examined using the Pearson deviance-to-degrees-of-freedom ratio, which consistently indicated slight underdispersion; therefore, robust (sandwich) variance estimators were retained. Linear regression assumptions—including linearity, normality and homoscedasticity of residuals, and influence diagnostics—were assessed using standard diagnostic plots, and no meaningful violations were identified. Linearity of continuous predictors, including IDDS and age, was further examined by comparing continuous and categorical model specifications.

Sensitivity analyses were conducted to assess the robustness of the findings. These included dichotomization of IDDS (high vs. low/medium), exclusion of participants with self-reported pre-existing conditions, and evaluation of potential IDDS × sex interaction terms. None of these analyses materially altered the magnitude or direction of the associations, and no interaction met the predefined threshold for meaningful effect modification (*p* < 0.10).

Missing data were minimal (<5%) and showed no evidence of systematic patterns; therefore, a complete-case analysis approach was adopted without imputation. All statistical tests were two-sided, and statistical significance was defined as *p* < 0.05.

## 3. Results

### 3.1. General Characteristics of the Study Population

A total of 349 university students were included in the final analysis. The median age was 21.5 years (interquartile range [IQR]: 20.0–23.0), reflecting a relatively homogeneous cohort of young adults enrolled in health sciences programs. Women comprised 67% of participants (*n* = 233), while men accounted for 33% (*n* = 116). The academic distribution largely mirrored the institutional composition: Medicine represented 38% of the sample (*n* = 132), followed by Nutrition and Dietetics (29%, *n* = 100), Physiotherapy (17%, *n* = 61), and Odontology (16%, *n* = 56). Nursing students accounted for fewer than 1% of participants and were therefore excluded from degree-stratified analyses due to insufficient sample size.

Lifestyle characteristics indicated that just over half of the students (51%) reported engaging in regular physical activity, whereas 49% reported insufficient activity levels. The prevalence of self-reported pre-existing health conditions was low (7%), consistent with a predominantly healthy young adult population.

Marked differences in lifestyle behaviors were observed by sex. Men were substantially more likely than women to report regular physical activity (70% vs. 41%, respectively; *p* < 0.001), whereas the prevalence of pre-existing health conditions did not differ between sexes (*p* = 0.934). Academic program distribution also varied by sex, with women more frequently enrolled in Nutrition and Dietetics and men disproportionately represented in Physiotherapy. These patterns highlight that sex and academic trajectory are closely related to differences in physical activity levels and may contribute to heterogeneity in body composition and cardiometabolic profiles observed in subsequent analyses.

### 3.2. Anthropometric and Body Composition Indicators

Anthropometric indicators were consistent with the profile of a young adult population but revealed a notable burden of excess adiposity. Median height was 1.61 m (IQR: 1.56–1.68), median body weight 63 kg (IQR: 55–75), and median BMI 24.3 kg/m^2^ (IQR: 21.7–27.3). Although more than half of the participants fell within the normal BMI range, a substantial proportion already met criteria for overweight or obesity, underscoring the early emergence of excess weight in this population.

Markers of fat distribution showed a median waist circumference of 76 cm (IQR: 69–83) and hip circumference of 98 cm (IQR: 92–104). The median WHR was 0.77 (IQR: 0.73–0.82), and the WHtR was 0.47 (IQR: 0.43–0.51), with a considerable proportion of students clustering near the WHtR ≥ 0.50 threshold that indicates elevated metabolic risk. Body composition values reflected a mixed adiposity–muscularity profile: median muscle mass was 22 kg (IQR: 19–30), fat mass 21 kg (IQR: 16–26), and body fat percentage 34% (IQR: 26–39). High or very high visceral adiposity was observed in 28% of participants, suggesting the presence of early metabolic vulnerability despite the young age of the cohort.

**Figure 1:**
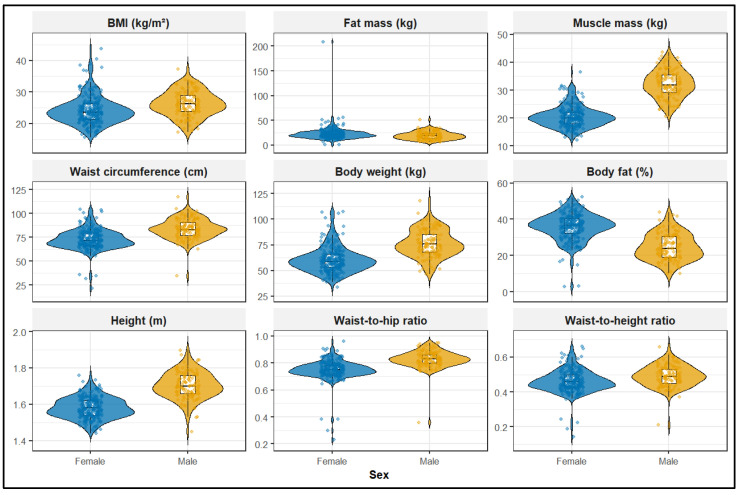
Sex-specific distributions of anthropometric and body composition indicators. Violin plots depict the distributions of selected anthropometric and body composition measures—BMI, fat mass, muscle mass, waist and hip circumferences, body weight, body fat percentage, height, WHR, and WHtR—stratified by sex. Boxplots indicate medians and interquartile ranges, with overlaid jittered points representing individual observations.

Pronounced sex-related differences were observed across nearly all indicators. Men exhibited significantly higher height (1.70 vs. 1.58 m), body weight (76 vs. 59 kg), and BMI (26.2 vs. 23.6 kg/m^2^), all with *p* < 0.001. Measures of central adiposity were consistently higher among men, including waist circumference (83 vs. 72 cm), hip circumference (101 vs. 96 cm), WHR (0.83 vs. 0.75), and WHtR (0.49 vs. 0.46) (all *p* < 0.001). In contrast, women demonstrated a more adipose but less centrally concentrated phenotype, with higher fat mass (21 vs. 19 kg; *p* = 0.001) and markedly higher body fat percentage (37% vs. 24%; *p* < 0.001), whereas men exhibited substantially greater muscle mass (32 vs. 20 kg; *p* < 0.001). Despite these divergences, the prevalence of high or very high visceral adiposity did not differ significantly by sex (*p* = 0.700), likely reflecting sex-specific reference values used in the device-derived estimates (Figure 1). Detailed sex-stratified anthropometric and body composition data are presented in Supplementary Table S1.

Physical activity status further differentiated anthropometric and body composition profiles. Students reporting regular physical activity displayed higher body weight, BMI, and muscle mass, accompanied by lower total fat mass and body fat percentage compared with insufficiently active peers. Differences in indicators of central adiposity were less consistent: waist circumference and WHR were higher among physically active students, whereas WHtR and visceral fat levels did not differ significantly between activity groups. These patterns likely reflect the greater representation of men among physically active participants and highlight the importance of accounting for sex and physical activity when interpreting adiposity-related indicators in young adults. A comprehensive comparison of anthropometric and body composition indicators by physical activity status is provided in Supplementary Table S2.

When comparing anthropometric characteristics across academic programs (excluding Nursing due to minimal sample size), significant differences were noted for most continuous variables. Height, weight, BMI, waist and hip circumferences, WHR, WHtR, muscle mass, and fat mass all varied across Medicine, Odontology, Nutrition and Dietetics, and Physiotherapy (all *p* ≤ 0.03). Nutrition and Dietetics students displayed lower median BMI and fat mass, suggesting healthier body composition patterns, whereas Physiotherapy students exhibited higher muscle mass and less favorable WHR profiles. Medical students tended to occupy intermediate positions, although they showed comparatively higher waist measurements despite similar BMI values. These program-level contrasts suggest that academic trajectory—likely reflecting underlying lifestyle behaviors, schedules, curricular demands, and sex composition—modulates body composition and adiposity profiles in meaningful ways.

### 3.3. Cardiometabolic Risk Indicators

Markers of cardiometabolic risk were already detectable in this cohort of young university students. Overall, 19% of participants met criteria for abdominal obesity, 12% presented elevated cardiovascular risk according to the waist-to-hip ratio (WHR), and 30% were classified as having metabolic risk based on the waist-to-height ratio (WHtR ≥ 0.50). Consequently, nearly one-third of the study population exhibited anthropometric profiles compatible with early metabolic vulnerability, despite their young age and the low prevalence of self-reported chronic conditions. These findings indicate that cardiometabolic risk factors may emerge earlier in the life course than traditionally assumed.

Sex-stratified analyses revealed distinct and partially divergent cardiometabolic risk profiles. Metabolic risk defined by WHtR ≥ 0.50 was significantly more prevalent among men than women (42% vs. 24%; *p* < 0.001), in line with their higher median waist circumference and WHtR values. In contrast, the prevalence of abdominal obesity, defined using sex-specific waist circumference cut-offs, did not differ significantly between men and women (*p* = 0.700). Cardiovascular risk assessed through WHR showed an inverse pattern, being more frequent among women than men (18% vs. 0%; *p* < 0.001). This apparent discrepancy reflects the application of sex-specific WHR thresholds, whereby women are classified at lower absolute WHR values, underscoring the necessity of interpreting these indicators within appropriate sex-specific reference frameworks rather than through direct numerical comparison. Sex-stratified distributions of cardiometabolic risk indicators are summarized in Supplementary Table S2.

When cardiometabolic risk indicators were examined across academic degree programs (excluding Nursing due to limited sample size), no statistically significant differences were observed in the prevalence of abdominal obesity or metabolic risk (*p* = 0.300 and *p* = 0.140, respectively). Nevertheless, students enrolled in Medicine and Physiotherapy tended to exhibit slightly higher proportions of metabolic risk compared with those studying Nutrition and Dietetics. In contrast, visceral adiposity classification differed significantly across programs (*p* = 0.002). Students from Nutrition and Dietetics showed the most favorable visceral fat profile, with 84% classified as having normal visceral adiposity, whereas higher proportions of elevated visceral fat levels were observed in the remaining programs. These program-level contrasts suggest that academic trajectory—potentially reflecting differences in sex composition, health-related knowledge, physical activity patterns, and daily routines—may influence the early accumulation of visceral fat, even in a predominantly young and ostensibly healthy population. Age- and health status–stratified distributions of anthropometric and body composition indicators are summarized in Supplementary Tables S3 and S4.

### 3.4. Dietary Diversity Indicators

The Individual Dietary Diversity Score (IDDS) showed a median value of 8.0 (IQR: 7.0–9.0), indicating that most students reported consumption from a relatively wide range of FAO-defined food groups. When categorized into tertiles, the distribution was balanced, with 32.6% of participants classified as having low dietary diversity, 30.6% as medium, and 36.8% as high. These proportions suggest that dietary diversity—understood as the number of distinct food groups consumed—was relatively homogeneous across the study population.

Consistent with this observation, IDDS tertiles did not differ significantly by sex (*p* = 0.113) or by academic degree program (*p* = 0.800), reinforcing the notion that dietary diversity, as operationalized by the IDDS, was broadly similar across demographic and academic strata. This homogeneity provides an appropriate context for examining whether variation in dietary diversity is associated with differences in anthropometric, behavioral, and cardiometabolic outcomes.

#### 3.4.1. Differences Across Tertiles of Dietary Diversity

Although sociodemographic characteristics were comparable across IDDS tertiles, several anthropometric and behavioral indicators varied meaningfully. Participants in the highest dietary diversity tertile exhibited a significantly higher median BMI compared with those in the lower tertiles (*p* = 0.027). Other markers of adiposity—including hip circumference, fat mass, and body fat percentage—also tended to be higher among individuals with greater dietary diversity, although these differences did not reach conventional thresholds for statistical significance (all *p* ≥ 0.095).

These patterns suggest that, within this cohort, higher dietary diversity may reflect broader overall food exposure rather than improved diet quality per se. Importantly, the IDDS captures the presence of food groups but does not account for consumption frequency, portion size, or energy density. As such, greater diversity may coexist with higher total energy intake or inclusion of energy-dense foods, particularly in settings where ultra-processed products are widely available.

Lifestyle behaviors showed clearer differentiation across dietary diversity levels. The prevalence of regular physical activity increased progressively across IDDS tertiles (41% in the low, 50% in the medium, and 59% in the high diversity group; *p* = 0.023), indicating a clustering of health-related behaviors among students with higher dietary diversity. In contrast, cardiometabolic risk indicators—including abdominal obesity, cardiovascular risk based on WHR, metabolic risk defined by WHtR ≥ 0.50, and visceral adiposity classification—did not differ significantly across dietary diversity categories (all *p* > 0.05). Collectively, these findings indicate that dietary diversity alone does not explain early cardiometabolic risk in this young adult population.

The internal consistency of the IDDS categorization was supported by the expected monotonic increase in IDDSs across tertiles (*p* < 0.001), confirming the robustness of this exposure variable for subsequent inferential and multivariable analyses.

#### 3.4.2. Differences Across Academic Degree Programs

Comparisons across academic degree programs (excluding Nursing due to limited sample size) revealed significant structuring of anthropometric and lifestyle characteristics. Continuous measures—including height, body weight, BMI, waist and hip circumferences, WHR, WHtR, muscle mass, and fat mass—differed significantly across students enrolled in Medicine, Odontology, Nutrition and Dietetics, and Physiotherapy (all *p* ≤ 0.03). Physiotherapy students tended to be taller, heavier, and more muscular, but also exhibited less favorable WHR and WHtR profiles. In contrast, students from Nutrition and Dietetics consistently displayed lower BMI and fat mass, along with more favorable indicators of central adiposity. Medical and Odontology students generally occupied intermediate positions across most anthropometric measures.

Among categorical variables, physical activity showed the most pronounced variation across programs (*p* = 0.006). Odontology students reported the lowest prevalence of regular physical activity (30%), whereas Medicine and Physiotherapy students reached prevalences of approximately 52–58%. BMI category distributions did not differ significantly across academic tracks (*p* = 0.344), with all programs showing substantial proportions of normal weight and mild-to-moderate overweight. Cardiovascular risk based on WHR was also similar across degrees (*p* = 0.500). In contrast, visceral adiposity classification varied significantly by program (*p* = 0.002), with Nutrition and Dietetics students again exhibiting the most favorable profile, characterized by the lowest prevalence of elevated visceral fat levels.

Taken together, these findings highlight that although dietary diversity is relatively homogeneous across sex and academic program groups, meaningful variation persists in anthropometric status, lifestyle behaviors, and visceral adiposity. This underscores the importance of considering academic trajectory, sex distribution, and behavioral patterns when interpreting dietary exposures and their associations with cardiometabolic and functional health outcomes. These descriptive results provide a solid empirical foundation for the multivariable analyses presented in the following section.

### 3.5. Food Group Consumption Patterns

Across the overall sample, food group consumption frequencies followed a characteristic pattern dominated by staple foods (G1), which constituted the most frequently consumed group (median 6.65 times/day; IQR: 4.41–9.54). Relatively high consumption was also observed for other vegetables (G10; median 3.15; IQR: 1.71–4.88), other fruits (G9; median 2.51; IQR: 2.72–7.11), and dairy products (G4; median 2.26; IQR: 2.21–4.07). Flesh foods (G5) were consumed at moderate levels (median 3.67; IQR: 2.41–6.79).

**Figure 2:**
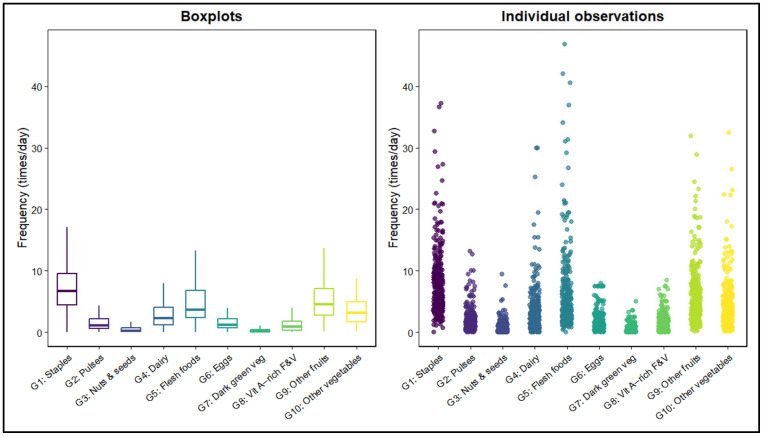
Distribution of FAO IDDS Food Group Consumption Frequencies. Boxplots (**left**) and individual observations (**right**) showing daily consumption frequencies of ten FAO IndiKit food groups. Groups represent standardized FAO categories summarizing related food items. Boxplots show medians and IQR without outliers; dots indicate raw values. Colors correspond to each food group.

In contrast, several food groups commonly considered nutrient-dense were consumed infrequently. These included pulses (G2; median 1.04), nuts and seeds (G3; median 0.18), dark green leafy vegetables (G7; median 0.12), and vitamin A–rich fruits and vegetables (G8; median 0.92). Together, these patterns indicate that students’ diets were largely centered on staple foods and commonly available produce, while the intake of plant-based protein sources, unsaturated fat sources, and carotenoid-rich foods remained comparatively low. The distribution of daily consumption frequencies across all FAO IndiKit food groups is illustrated in Figure 2.

When food group consumption was compared across academic degree programs (excluding Nursing due to limited sample size), most food groups showed similar intake levels across Medicine, Nutrition and Dietetics, Odontology, and Physiotherapy students. No statistically significant differences were observed for staples (G1), pulses (G2), nuts and seeds (G3), flesh foods (G5), eggs (G6), dark green leafy vegetables (G7), other vegetables (G10), or other fruits (G9) (all *p* > 0.10). These findings indicate a broadly homogeneous dietary pattern across academic tracks with respect to the majority of FAO-defined food groups.

However, two food groups exhibited significant program-level variation. Dairy consumption (G4) differed significantly across programs (*p* = 0.038), with the highest median intake reported among students in Medicine and Nutrition and Dietetics (2.58 and 2.32 times/day, respectively), intermediate consumption among Physiotherapy students, and the lowest intake among Odontology students (1.46 times/day). In addition, intake of vitamin A–rich fruits and vegetables (G8) varied significantly by academic program (*p* = 0.047). Students enrolled in Nutrition and Dietetics reported the highest median consumption (1.00 times/day), followed by those in Physiotherapy and Medicine, whereas Odontology students exhibited the lowest intake (0.52 times/day). Although not statistically significant, Physiotherapy students showed a tendency toward slightly higher consumption of other fruits (G9; median 3.6 times/day) compared with the remaining programs (2.7–3.2 times/day). Intake of other vegetables (G10) remained consistently moderate to high across all academic tracks.

Overall, food group consumption patterns were largely similar across degree programs; nevertheless, students from Nutrition and Dietetics demonstrated a comparatively more nutrient-dense profile, particularly with respect to dairy products and vitamin A–rich foods. In contrast, Odontology students consistently reported the lowest intake of these micronutrient-rich groups, while Physiotherapy students tended to consume fruits more frequently. These program-level differences, although modest, complement the anthropometric and lifestyle variations previously described and suggest that academic pathway and associated behaviors may shape subtle yet meaningful distinctions in habitual food group consumption within this young adult population.

### 3.6. Bivariable Associations Based on Dietary Diversity and Data-Driven Dietary Patterns

In bivariable analyses, dietary diversity assessed as a continuous exposure using the IDDS was positively associated with excess body weight. Each one-unit increase in IDDS was associated with an 18% higher prevalence of excess weight (PR = 1.18; 95% CI: 1.07–1.30; *p* = 0.001). Although dietary diversity is often interpreted as a proxy for dietary adequacy, this direction of association is compatible with the notion that, in this young adult university population, higher dietary diversity may reflect broader overall food exposure rather than healthier dietary profiles per se. Given that the IDDS captures the presence of food groups without weighting by portion size, frequency, or energy density, the underlying drivers of this association cannot be disentangled within the present cross-sectional design.

No significant association was observed between IDDS and low handgrip strength (PR = 0.93; 95% CI: 0.86–1.02), indicating that crude dietary diversity was not related to differences in muscular performance. This finding is consistent with the multifactorial determinants of muscle strength in young adults, in whom sex, neuromuscular adaptation, and physical activity levels may exert a stronger influence than dietary variety alone in unadjusted analyses. In contrast, IDDS showed a positive association with relative muscle mass (β = 0.261; 95% CI: 0.130–0.392; *p* < 0.001), suggesting that greater dietary diversity was associated with higher lean mass relative to body size. This relationship should be interpreted as an association with body composition rather than as evidence of improved muscle function or quality.

When dietary diversity was categorized into tertiles, the direction and magnitude of associations were consistent with those observed in the continuous models. Participants in the highest IDDS tertile exhibited a significantly higher prevalence of excess weight compared with those in the lowest tertile (PR = 1.60; 95% CI: 1.18–2.18; *p* = 0.002), whereas no significant differences were observed for the medium-tertile group. Associations with relative muscle mass also remained robust, with both medium- and high-diversity tertiles showing higher values compared with the low-diversity reference group. Across all tertiles, no significant associations were identified between dietary diversity and low handgrip strength, reinforcing the absence of a crude relationship between dietary variety and functional strength outcomes.

Bivariable analyses based on data-driven dietary patterns, derived through principal component analysis followed by k-means clustering, did not reveal significant associations with any of the study outcomes. Neither the Energy-Dense/Ultra-Processed pattern nor the Prudent/Whole-Food pattern showed meaningful relationships with excess body weight, low handgrip strength, or relative muscle mass. Effect estimates were consistently close to the null. These findings likely reflect limited exposure contrast within dietary patterns in this relatively homogeneous and metabolically young population, rather than a lack of relevance of dietary pattern analysis per se.

The divergence between IDDS-based associations and those derived from PCA-based dietary patterns highlights an important methodological distinction. The IDDS captures the breadth of food group consumption, which may indirectly correlate with overall intake volume and body size, whereas PCA-derived patterns emphasize the covariance structure of dietary behaviors and may attenuate inter-individual variability when dietary habits are broadly similar across participants. In this context, the absence of significant associations for PCA-derived patterns provides complementary information, indicating that while dietary diversity varies sufficiently to show crude associations with body composition, broader multivariate dietary patterns may not be strongly differentiated in this cohort. Complete results of bivariable analyses for dietary diversity and data-driven dietary patterns are reported in Supplementary Tables S5 and S6.

### 3.7. Multivariable Associations Between Dietary Exposures and Health Indicators

In multivariable Poisson regression models adjusted for age, sex, academic degree program, physical activity, and self-reported pre-existing conditions, dietary diversity assessed by the IDDS was not independently associated with low handgrip strength. Adjusted prevalence ratios remained close to the null across all model specifications, indicating that dietary diversity alone was not related to functional muscle strength in this young adult population.

**Figure 3:**
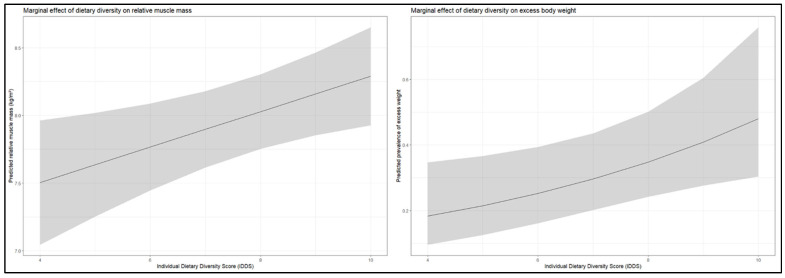
Adjusted Marginal Effects of Dietary Diversity on Excess Body Weight and Relative Muscle Mass. Combined marginal effect plots showing the adjusted predicted prevalence of excess body weight and the adjusted predicted relative muscle mass across the range of IDDS. Shaded bands represent 95% confidence intervals obtained from multivariable Poisson and linear regression models adjusted for age, sex, academic program, physical activity, and pre-existing health conditions.

**Figure 4:**
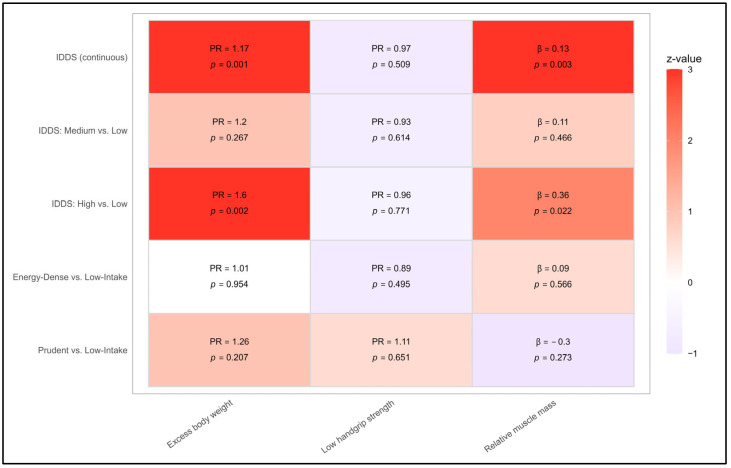
Heatmap of adjusted associations between dietary exposures and health indicators. Heatmap summarizing adjusted effect estimates for associations between dietary exposures—IDDS (continuous and tertiles) and PCA-derived patterns—and three outcomes: excess body weight, low handgrip strength, and relative muscle mass. Colors represent standardized z-values, with red indicating stronger positive associations and purple indicating weaker or null associations. Statistical significance markers (PRs or β coefficients with *p*-values) are embedded within each cell. Significant associations were observed only for IDDS measures in relation to excess body weight and relative muscle mass.

For excess body weight, IDDS modeled as a continuous exposure retained a modest but statistically significant association after adjustment (PR = 1.17; 95% CI: 1.06–1.30). Although this finding indicates that higher dietary diversity was associated with a greater prevalence of excess weight, the magnitude of the association was small and no clear linear dose–response relationship was observed. Consistent with this pattern, when IDDS was categorized into tertiles, only participants in the highest dietary diversity tertile exhibited a significantly higher adjusted prevalence of excess weight compared with those in the lowest tertile (PR = 1.60; 95% CI: 1.19–2.14), whereas the medium-diversity group did not differ significantly. This pattern supports the presence of a threshold-type association rather than a graded gradient across the full range of dietary diversity (Figure 3 and Figure 4).

With respect to relative muscle mass, IDDS remained positively associated in the adjusted continuous model (β = 0.131; 95% CI: 0.046–0.216), indicating that higher dietary diversity was independently associated with greater lean mass relative to body size. However, analyses based on tertiles showed that this association was primarily driven by participants in the highest dietary diversity group, again suggesting a threshold effect rather than a uniform increase across all levels of dietary diversity (Figure 3 and Figure 4). As in the bivariable analyses, these associations pertain to body composition and should not be interpreted as evidence of improved muscle function.

In contrast, PCA-derived dietary patterns did not demonstrate independent associations with excess body weight, low handgrip strength, or relative muscle mass after covariate adjustment. Effect estimates for both the Energy-Dense/Ultra-Processed and Prudent/Whole-Food patterns remained centered around the null across all outcomes. These findings are consistent with the limited dietary exposure contrast observed within the cohort and with the relative homogeneity of dietary behaviors in this metabolically young population.

Covariate effects across multivariable models were internally consistent and biologically plausible. Male sex and older age were associated with higher relative muscle mass, while regular physical activity was inversely associated with low handgrip strength and positively related to lean mass, independent of dietary exposures. Academic degree programs with very small sample sizes, such as Nursing, yielded unstable estimates and were therefore excluded from program-specific interpretation. Fully adjusted multivariable regression models, are presented in Supplementary Tables S7 and S8.

### 3.8. Model Assumptions and Diagnostic Evaluation

Comprehensive diagnostic procedures indicated satisfactory performance of all multivariable models. Generalized variance inflation factors (GVIF-adjusted) were below 1.08 for all predictors, indicating negligible multicollinearity and stable coefficient estimation across model specifications. Poisson regression models exhibited slight underdispersion (Pearson deviance-to-degrees-of-freedom ratio ≈ 0.55–0.57), a pattern that does not compromise model validity and further supports the use of robust (sandwich) variance estimators to ensure reliable standard error estimation.

For the linear regression model assessing relative muscle mass, diagnostic evaluations demonstrated adequate adherence to model assumptions, including approximate normality of residuals, homoscedasticity, and linearity. One high-leverage observation was identified; however, its exclusion did not materially alter coefficient estimates or confidence intervals, indicating minimal influence on model inference. Linearity of continuous predictors, including IDDS and age, was further verified through comparison with alternative categorical and spline-like parameterizations, with no evidence of improved model fit.

Sensitivity analyses supported the stability of the main findings. Exclusion of participants with self-reported pre-existing conditions, alternative specification of dietary diversity (high versus low/medium IDDS), and evaluation of potential IDDS × sex interactions did not result in meaningful changes in effect magnitude or statistical significance. No interaction term met the predefined threshold for effect modification (*p* < 0.10), indicating that observed associations were consistent across men and women.

## 4. Discussion

In this cross-sectional study of Ecuadorian university students, several relevant findings emerged. Despite the young age of the participants and the low prevalence of diagnosed chronic conditions, a substantial proportion already exhibited markers of early cardiometabolic risk, including excess body weight, central adiposity, and elevated waist-to-height ratio (WHtR). These findings align with accumulating evidence indicating that cardiometabolic risk factors can be detected in young populations, even before clinically overt disease becomes common [2,41,42]. In this context, screening approaches based on central adiposity appear particularly informative. WHtR has been proposed as a practical and sensitive marker of cardiometabolic risk in younger age groups, with a cutoff around 0.5 frequently discussed as a simple and population-applicable threshold [43,44,45].

Dietary diversity, assessed using the FAO Individual Dietary Diversity Score (IDDS), showed a dual pattern of association in this study. Higher dietary diversity was positively associated with both excess body weight and relative muscle mass, whereas no association was observed with handgrip strength. These findings are consistent with the broader literature showing that the relationship between dietary diversity and adiposity is heterogeneous. A systematic review concluded that associations between dietary diversity scores and obesity vary substantially across observational studies, depending on population characteristics, scoring methods, and food environments [46]. Importantly, an American Heart Association Science Advisory emphasized that the available evidence does not support increasing dietary diversity as a standalone strategy for promoting healthy body weight, as “diversity” may encompass both healthy and unhealthy foods and may be accompanied by higher total energy intake [21]. This interpretation is consistent with contemporary analyses indicating that more varied diets can coexist with higher energy intake, even in the absence of consistent differences in BMI across settings [14,22].

At the same time, the positive association between dietary diversity and relative muscle mass observed in our cohort is biologically plausible but warrants careful interpretation. Dietary diversity metrics increase the likelihood of exposure to a broader range of nutrient sources relevant to body composition; however, they do not capture the quality, quantity, or bioavailability of specific nutrients. Evidence from Latin America supports the notion that higher dietary diversity, operationalized through minimum diversity metrics, is associated with improved micronutrient adequacy—an upstream factor that may contribute to differences in body composition without necessarily translating into functional benefits [15].

Importantly, evidence from body composition research indicates that higher estimates of skeletal muscle mass—particularly when assessed using bioelectrical impedance analysis (BIA)—are frequently observed in individuals with greater overall body mass or fat mass. For example, muscle thickness assessed by ultrasonography has been shown to correlate more strongly with muscle strength and quality than appendicular skeletal muscle index (ASMI) derived from BIA, especially in non-overweight individuals, pointing to limitations of BIA in reflecting functional muscle properties rather than body size effects [33]. Moreover, comparisons between BIA and dual-energy X-ray absorptiometry (DXA) indicate that BIA tends to overestimate skeletal muscle mass relative to more precise methods, which can mask differences in muscle function or quality when used alone [47]. Likewise, reviews of BIA methodology highlight that its estimations may not fully capture muscle quality or performance, particularly in the presence of higher fat mass, reinforcing the notion that greater estimated muscle mass does not necessarily translate into superior muscle strength or capacity [48].

In contrast, the absence of an association between dietary diversity and handgrip strength is compatible with existing evidence suggesting that muscle strength in young adults is strongly influenced by physical activity, sex-related physiological differences, and neuromuscular adaptation. Greater muscle mass does not necessarily imply improved muscle quality or neuromuscular performance, particularly when muscle composition is assessed using bioelectrical impedance rather than direct functional or structural measures. Diet–strength associations may therefore be modest or context dependent in generally healthy, young populations [28,49]

Dietary patterns derived using principal component analysis (PCA) did not show significant associations with adiposity, muscular strength, or relative muscle mass after multivariable adjustment. This finding should be interpreted in light of the methodological characteristics of PCA-based approaches. PCA summarizes dietary intake by extracting components based on the correlation or covariance structure of foods or food groups, with the primary objective of characterizing prevailing dietary structures within a given population rather than maximizing exposure–outcome contrasts [50,51]. Methodological work has shown that PCA-derived patterns are sensitive to analytic decisions such as food grouping, scaling, and the number of components retained, and that they primarily reflect the dietary variability present within the specific study sample [26,27,51,52,53]. Consequently, in relatively homogeneous populations—such as university students enrolled in similar academic programs and sharing comparable food environments—PCA may offer limited exposure contrast for detecting associations with health outcomes. This provides a plausible explanation for the null pattern–outcome findings observed in our study, despite detectable associations when dietary diversity was assessed using the IDDS [26,27].

Our results should also be interpreted within the broader regional context of ongoing nutrition transition and the rising availability and consumption of ultra-processed foods in Latin America, as documented by the Pan American Health Organization [34]. In this context, evidence from university students across multiple Latin American countries indicates that higher consumption of ultra-processed foods is associated with overweight and obesity, supporting the notion that dietary variety in modern food environments can coexist with higher energy density and adverse weight outcomes [54].

This study has several strengths. It directly compared two commonly used operationalizations of diet—dietary diversity and PCA-derived dietary patterns—within the same cohort, allowing an internally consistent evaluation of how these constructs relate to adiposity and muscular outcomes. The inclusion of multidimensional outcomes, encompassing anthropometry, body composition, and muscular function, enhances interpretability beyond BMI alone. Moreover, focusing on young adults in Ecuador contributes to addressing a documented gap in nutritional epidemiology among Latin American university-age populations.

Several limitations should also be acknowledged. The cross-sectional design precludes causal inference, and dietary intake was self-reported, which may introduce recall bias. The use of IDDS entails conceptual constraints, as the score does not account for food sources, portion size, degree of processing, or preparation methods. As a result, nutritionally distinct foods—such as minimally processed and ultra-processed items within the same food group—are weighted equally, which may limit interpretation in contemporary urban food environments. Additionally, PCA-derived dietary patterns are sample dependent and sensitive to analytic decisions, and limited dietary variability in this relatively homogeneous population may have reduced the ability to detect associations. Finally, the single-institution sample may limit generalizability to other young adult populations.

From a clinical perspective, these findings suggest that higher dietary diversity should not be interpreted as inherently protective in young adults. Recommendations promoting dietary variety may benefit from being coupled with explicit emphasis on food quality, degree of processing, and energy density, given that dietary diversity can coexist with higher energy intake and excess body weight in modern food environments. At the same time, the observed association between dietary diversity and relative muscle mass supports counseling strategies that encourage nutrient-dense variety, rather than diversity per se, particularly in the context of adequate physical activity.

Universities represent a strategic setting for early prevention, as cardiometabolic risk markers can emerge during young adulthood. Public health strategies may therefore benefit from moving beyond generic “eat a varied diet” messaging and incorporating structural actions that improve campus food environments and reduce exposure to ultra-processed foods, particularly in Latin America, where growth in ultra-processed food availability has been well documented.

Finally, longitudinal studies are needed to clarify the temporal relationships between dietary diversity, dietary patterns, and trajectories of adiposity and muscular health from university years into adulthood. Future research should integrate diversity metrics with indicators of food processing level and energy density to disentangle “healthy diversity” from “obesogenic diversity.” Methodologically, studies in relatively homogeneous young adult populations should explore alternative pattern-derivation approaches, such as reduced rank regression or a priori dietary indices, alongside PCA to enhance exposure contrast and robustness. Expanding research across diverse Latin American university contexts will further strengthen external validity and support the development of feasible, population-appropriate prevention strategies in the context of rapid nutrition transition.

## 5. Conclusions

In Ecuadorian university students, markers of cardiometabolic risk were already prevalent despite young age and a low burden of diagnosed disease. Dietary diversity, assessed using the FAO Individual Dietary Diversity Score, showed a dual pattern of association, being positively related to both excess body weight and relative muscle mass, while no association was observed with muscular strength. Importantly, the association with greater muscle mass did not translate into improved functional performance, underscoring the distinction between muscle quantity and muscle function in young adults. In contrast, dietary patterns derived through principal component analysis were not associated with adiposity or functional outcomes after multivariable adjustment.

Together, these findings suggest that dietary diversity reflects the breadth of food group consumption rather than dietary quality per se and may coexist with excess body weight in contemporary food environments. From a preventive perspective, these results highlight the importance of moving beyond generic recommendations for dietary variety in young adults and instead emphasizing food quality, degree of processing, and nutrient-dense dietary choices when addressing early cardiometabolic risk, particularly in settings undergoing rapid nutrition transition.

## Data Availability

The datasets analyzed during the current study are available from the corresponding author upon reasonable request. Due to participant confidentiality and institutional data protection policies, individual-level data cannot be shared publicly. Aggregated and de-identified data supporting the findings of this study will be provided upon justified request for academic or research purposes.

## Supplementary Materials

The following supporting information can be downloaded at: https://www.mdpi.com/article/10.3390/nu18030511/s1, Table S1: Anthropometric and body composition indicators stratified by sex. Table S2: Anthropometric and body composition indicators stratified by physical activity status. Table S3: Anthropometric and body composition indicators stratified by age group. Table S4: Anthropometric and body composition indicators stratified by pre-existing conditions. Table S5: Crude associations between the Individual Dietary Diversity Score (IDDS) and excess body weight, low handgrip strength, and relative muscle mass among university students. Table S6: Crude associations between data-driven dietary patterns and excess body weight, low handgrip strength, and relative muscle mass. Table S7: Adjusted associations between the Individual Dietary Diversity Score (IDDS) and excess body weight, low handgrip strength, and relative muscle mass. Table S8: Adjusted associations between PCA-derived dietary patterns and excess body weight, low handgrip strength, and relative muscle mass.
