# Supplementary material for: Dietary Diversity, Dietary Patterns, and Cardiometabolic Health in University Students: A Cross-Sectional Study
Source: Nutrients. 2026 Feb 2;18(3):511. doi: 10.3390/nu18030511 (PMC12899775; doi:10.3390/nu18030511)
Supplement: Supplementary file 1 [file nutrients-18-00511-s001.zip › nutrients-4094772-supplementary.pdf]

Supplementary Material

Supplementary Tables

Table S1. Anthropometric and body composition indicators stratified by sex

Characteristic	Female (N = 232)	Male (N = 117)	p-value[2]
**Weight, kg**	59 [53, 66]	76 [68, 85]	<0.001
**BMI, kg/m^2**	23.6 [21.4, 26.3]	26.2 [23.9, 29.0]	<0.001
**Waist circumference, cm**	72 [67, 79]	83 [77, 91]	<0.001
**Hip circumference, cm**	96 [91, 102]	101 [96, 107]	<0.001
**Waist-to-hip ratio (WHR)**	0.75 [0.72, 0.78]	0.83 [0.80, 0.86]	<0.001
**Waist-to-height ratio (WHtR)**	0.46 [0.43, 0.50]	0.49 [0.45, 0.53]	<0.001
**Muscle mass, kg**	20 [18, 22]	32 [29, 36]	<0.001
**Fat mass, kg**	21 [17, 27]	19 [13, 24]	0.001
**Body fat, %**	37 [32, 41]	24 [19, 30]	<0.001
**Height, m**	1.58 [1.54, 1.62]	1.70 [1.66, 1.76]	<0.001
**Visceral fat level**	8.0 [6.0, 10.0]	7.0 [5.0, 10.0]	0.035
**Degree program, n (%)**			<0.001
Medicine	84 (36%)	48 (41%)	
Odontology	39 (17%)	17 (15%)	
Nutrition and Dietetics	80 (34%)	20 (17%)	
Physiotherapy	29 (13%)	32 (27%)	
**Pre-existing conditions, n (%)**			>0.9
No	214 (92%)	109 (93%)	
Yes	18 (7.8%)	8 (6.8%)	

**Physical activity, n (%)**			<0.001
Insufficient	137 (59%)	35 (30%)	
Regular	95 (41%)	82 (70%)	
**BMI category, n (%)**			<0.001
Underweight	15 (6.5%)	4 (3.4%)	
Normal weight	137 (59%)	41 (35%)	
Overweight I	39 (17%)	22 (19%)	
Overweight II	18 (7.8%)	27 (23%)	
Obesity I	16 (6.9%)	22 (19%)	
Obesity II	5 (2.2%)	1 (0.9%)	
Obesity III	2 (0.9%)	0 (0%)	
**Abdominal obesity, n (%)**			0.8
No	186 (80%)	96 (82%)	
Yes	46 (20%)	21 (18%)	
**Cardiovascular risk (WHR), n (%)**			<0.001
No	190 (82%)	117 (100%)	
Yes	42 (18%)	0 (0%)	
**Metabolic risk (WHtR ≥ 0.50), n (%)**			<0.001
No	177 (76%)	68 (58%)	
Yes	55 (24%)	49 (42%)	
**Visceral fat category, n (%)**			0.6
Normal	166 (72%)	86 (74%)	
High	58 (25%)	29 (25%)	
Very high	8 (3.4%)	2 (1.7%)	

Values are median [Q1, Q3] or n (%). [2] Wilcoxon rank-sum test; Pearson’s Chi-squared test.

Table S2. Anthropometric and body composition indicators stratified by physical activity status

Characteristic	Insufficient(N = 172)	Regular(N = 177)	p-value[1]
**Weight, kg**	60 [53, 70]	67 [57, 77]	<0.001
**BMI, kg/m²**	23.8 [21.5, 26.6]	24.7 [22.4, 27.7]	0.035
**Waist circumference, cm**	74 [68, 81]	78 [70, 86]	0.008
**Hip circumference, cm**	98 [91, 103]	99 [93, 105]	0.124
**Waist-to-hip ratio (WHR)**	0.76 [0.73, 0.81]	0.79 [0.75, 0.83]	0.001
**Waist-to-height ratio (WHtR)**	0.46 [0.43, 0.51]	0.47 [0.43, 0.51]	0.181
**Muscle mass, kg**	21 [18, 24]	25 [21, 32]	<0.001
**Fat mass, kg**	22 [17, 27]	19 [15, 25]	0.021
**Body fat, %**	36 [30, 40]	30 [23, 37]	<0.001
**Visceral fat level**	8.0 [6.0, 10.0]	7.0 [6.0, 9.0]	0.080
**Sex, n (%)**			<0.001
Female	137 (80%)	95 (54%)	
Male	35 (20%)	82 (46%)	
**Degree program, n (%)**			0.006
Medicine	55 (32%)	77 (44%)	
Odontology	39 (23%)	17 (9.6%)	
Nutrition and Dietetics	48 (28%)	52 (29%)	
Physiotherapy	30 (17%)	31 (18%)	
**Pre-existing conditions, n (%)**			0.345
No	162 (94%)	161 (91%)	
Yes	10 (5.8%)	16 (9.0%)	
**BMI category, n (%)**			0.370

Underweight	11 (6.4%)	8 (4.5%)	
Normal weight	93 (54%)	85 (48%)	
Overweight I	33 (19%)	28 (16%)	
Overweight II	17 (9.9%)	28 (16%)	
Obesity I	14 (8.1%)	24 (14%)	
Obesity II	3 (1.7%)	3 (1.7%)	
Obesity III	1 (0.6%)	1 (0.6%)	
**Abdominal obesity, n (%)**			0.679
No	141 (82%)	141 (80%)	
Yes	31 (18%)	36 (20%)	
**Cardiovascular risk (WHR), n (%)**			0.357
No	148 (86%)	159 (90%)	
Yes	24 (14%)	18 (10%)	
**Metabolic risk (WHtR ≥ 0.50), n (%)**			0.379
No	125 (73%)	120 (68%)	
Yes	47 (27%)	57 (32%)	
**Visceral fat category, n (%)**			0.311
Normal	118 (69%)	134 (76%)	
High	49 (28%)	38 (21%)	
Very high	5 (2.9%)	5 (2.8%)	

[1] Wilcoxon rank-sum test for continuous variables; Pearson's Chi-squared test for categorical variables.

Table S3. Anthropometric and body composition indicators stratified by age group

Characteristic	18–20 years (N = 120)	21–23 years (N = 169)	24–26 years (N = 60)	p-value[2]
**Weight, kg**	61 [52, 72]	63 [56, 75]	68 [60, 80]	0.009
**BMI, kg/m²**	23.7 [20.7, 26.4]	24.5 [22.4, 27.3]	26.1 [23.5, 29.0]	<0.001
**Waist circumference, cm**	74 [67, 80]	75 [70, 83]	81 [72, 87]	0.001
**Hip circumference, cm**	96 [90, 102]	99 [93, 104]	101 [95, 107]	0.005
**Waist-to-hip ratio (WHR)**	0.76 [0.73, 0.80]	0.78 [0.74, 0.82]	0.80 [0.73, 0.85]	0.025
**Waist-to-height ratio (WHtR)**	0.46 [0.42, 0.49]	0.47 [0.44, 0.51]	0.49 [0.45, 0.53]	<0.001
**Muscle mass, kg**	21 [19, 28]	23 [20, 30]	23 [20, 31]	0.200
**Fat mass, kg**	19 [15, 24]	20 [16, 26]	23 [18, 28]	0.017
**Body fat, %**	33 [25, 37]	34 [26, 39]	35 [27, 40]	0.300
**Height, m**	1.62 [1.56, 1.67]	1.61 [1.56, 1.69]	1.62 [1.55, 1.69]	>0.9
**Visceral fat level**	7.0 [5.0, 9.0]	8.0 [6.0, 10.0]	8.5 [6.0, 11.0]	0.045
**Sex, n (%)**				0.5
Female	85 (71%)	108 (64%)	34 (65%)	
Male	35 (29%)	61 (36%)	18 (35%)	
**Degree program, n (%)**				<0.001
Medicine	59 (49%)	63 (37%)	10 (19%)	
Odontology	21 (18%)	18 (11%)	16 (31%)	
Nutrition and Dietetics	32 (27%)	47 (28%)	17 (33%)	
Physiotherapy	8 (6.7%)	41 (24%)	9 (17%)	
**Pre-existing conditions, n (%)**				0.086
No	116 (96.7%)	153 (90.5%)	46 (88%)	
Yes	4 (3.3%)	16 (9.5%)	6 (12%)	

**Physical activity, n (%)**				0.2
Insufficient	67 (56%)	77 (46%)	23 (44%)	
Regular	53 (44%)	92 (54%)	29 (56%)	
**BMI category, n (%)**				
Underweight	14 (12%)	3 (1.8%)	2 (3.8%)	
Normal weight	64 (53%)	93 (55%)	18 (35%)	
Overweight I	20 (17%)	30 (18%)	11 (21%)	
Overweight II	14 (12%)	20 (12%)	10 (19%)	
Obesity I	7 (5.8%)	18 (11%)	9 (17%)	
Obesity II	0 (0%)	5 (3.0%)	1 (1.9%)	
Obesity III	1 (0.8%)	0 (0%)	1 (1.9%)	
**Abdominal obesity, n (%)**				<0.001
No	105 (88%)	140 (83%)	33 (63%)	
Yes	15 (13%)	29 (17%)	19 (37%)	
**Cardiovascular risk (WHR), n (%)**				0.2
No	110 (92%)	146 (86%)	43 (83%)	
Yes	10 (8.3%)	23 (14%)	9 (17%)	
**Metabolic risk (WHtR ≥ 0.50), n (%)**				0.011
No	94 (78%)	118 (70%)	29 (56%)	
Yes	26 (22%)	51 (30%)	23 (44%)	
**Visceral fat category, n (%)**				0.7
Normal	91 (76%)	123 (73%)	34 (65%)	
High	27 (23%)	41 (24%)	16 (31%)	
Very high	2 (1.7%)	5 (3.0%)	2 (3.8%)	

Values are median [Q1, Q3] or n (%). [2] Kruskal–Wallis rank-sum test; Pearson's Chi-squared test with simulated p-value (20,000 replicates).

Table S4. Anthropometric and body composition indicators stratified by pre-existing conditions

Characteristic	No (N = 323)	Yes (N = 26)	p-value²
**Weight, kg**	63 [55, 74]	64 [57, 75]	0.6
**BMI, kg/m²**	24.3 [21.8, 27.2]	24.1 [21.4, 28.0]	0.9
**Waist circumference, cm**	76 [69, 83]	76 [68, 86]	>0.9
**Hip circumference, cm**	98 [92, 104]	99 [92, 105]	0.8
**Waist-to-hip ratio (WHR)**	0.78 [0.73, 0.82]	0.75 [0.73, 0.82]	0.2
**Waist-to-height ratio (WHtR)**	0.47 [0.43, 0.51]	0.46 [0.43, 0.52]	0.9
**Muscle mass, kg**	22 [19, 30]	24 [19, 31]	0.5
**Fat mass, kg**	21 [16, 26]	18 [16, 27]	0.7
**Body fat, %**	34 [26, 39]	35 [22, 38]	0.7
**Height, m**	1.61 [1.56, 1.68]	1.62 [1.53, 1.68]	0.8
**Visceral fat level**	8.0 [6.0, 10.0]	7.0 [6.0, 10.0]	0.5
**Sex, n (%)**			>0.9
Female	214 (66%)	18 (69%)	
Male	109 (34%)	8 (31%)	
Degree program, n (%)			0.6
Medicine	121 (37%)	11 (42%)	
Odontology	54 (17%)	2 (7.7%)	
Nutrition and Dietetics	91 (28%)	9 (35%)	
Physiotherapy	57 (18%)	4 (15%)	
**Physical activity, n (%)**			0.3
Insufficient	162 (50%)	10 (38%)	
Regular	161 (50%)	16 (62%)	

**BMI category, n (%)**			0.2
Underweight	19 (5.9%)	0 (0%)	
Normal weight	163 (50%)	15 (58%)	
Overweight I	58 (18%)	3 (12%)	
Overweight II	42 (13%)	3 (12%)	
Obesity I	35 (11%)	3 (12%)	
Obesity II	5 (1.5%)	1 (3.8%)	
Obesity III	1 (0.3%)	1 (3.8%)	
**Abdominal obesity, n (%)**			0.8
No	262 (81%)	20 (77%)	
Yes	61 (19%)	6 (23%)	
**Cardiovascular risk (WHR), n (%)**			0.7
No	283 (88%)	24 (92%)	
Yes	40 (12%)	2 (7.7%)	
**Metabolic risk (WHtR ≥ 0.50), n (%)**			>0.9
No	227 (70%)	18 (69%)	
Yes	96 (30%)	8 (31%)	
**Visceral fat category, n (%)**			0.3
Normal	233 (72%)	19 (73%)	
High	82 (25%)	5 (19%)	
Very high	8 (2.5%)	2 (7.7%)	

Values are median [Q1, Q3] or n (%).

[2] Wilcoxon rank-sum test; Pearson's Chi-squared test.

Table S5. Crude associations between the Individual Dietary Diversity Score (IDDS) and excess body weight, low handgrip strength, and relative muscle mass among university students.

Exposure	Outcome	Effect estimate	95% CI	p-value
IDDS (continuous)	Excess body weight	PR = 1.18	1.07–1.30	0.001
IDDS (continuous)	Low handgrip strength	PR = 0.93	0.86–1.02	0.109
IDDS (continuous)	Relative muscle mass	β = 0.261	0.130–0.392	<0.001
Medium IDDS tertile vs. Low	Excess body weight	PR = 1.20	0.87–1.66	0.267
High IDDS tertile vs. Low	Excess body weight	PR = 1.60	1.18–2.18	0.002
Medium IDDS tertile vs. Low	Low handgrip strength	PR = 0.93	0.71–1.22	0.614
High IDDS tertile vs. Low	Low handgrip strength	PR = 0.96	0.74–1.25	0.771
Medium IDDS tertile vs. Low	Relative muscle mass	β = 0.109	−0.184–0.401	0.466
High IDDS tertile vs. Low	Relative muscle mass	β = 0.365	0.054–0.676	0.022

Footnote: PR, prevalence ratio; β, linear regression coefficient. Crude Poisson regression with robust variance was used for binary outcomes, and linear regression for relative muscle mass.

Table S6. Crude associations between data-driven dietary patterns and excess body weight, low handgrip strength, and relative muscle mass.

Dietary pattern (reference: Low-Intake)	Outcome	Effect estimate	95% CI	p-value
Energy-Dense/Ultra-Processed	Excess body weight	PR = 1.01	0.74–1.38	0.954
Prudent/Whole-Food	Excess body weight	PR = 1.26	0.88–1.80	0.207
Energy-Dense/Ultra-Processed	Low handgrip strength	PR = 0.89	0.63–1.25	0.495
Prudent/Whole-Food	Low handgrip strength	PR = 1.11	0.70–1.78	0.651
Energy-Dense/Ultra-Processed	Relative muscle mass	β = 0.094	−0.226–0.413	0.566
Prudent/Whole-Food	Relative muscle mass	β = −0.297	−0.826–0.233	0.273

Footnote: No statistically significant crude associations were observed for PCA-derived dietary patterns.

Table S7. Adjusted associations between the Individual Dietary Diversity Score (IDDS) and excess body weight, low handgrip strength, and relative muscle mass.

Exposure	Outcome	Adjusted effect	95% CI	p-value
IDDS (continuous)	Excess body weight	PR = 1.17	1.06–1.30	0.001
IDDS (continuous)	Low handgrip strength	PR = 0.97	0.90–1.06	0.509
IDDS (continuous)	Relative muscle mass	β = 0.131	0.046–0.216	0.003
Medium IDDS tertile vs. Low	Excess body weight	PR = 1.20	0.87–1.66	0.267
High IDDS tertile vs. Low	Excess body weight	PR = 1.60	1.19–2.14	0.002
Medium IDDS tertile vs. Low	Low handgrip strength	PR = 0.93	0.71–1.22	0.614
High IDDS tertile vs. Low	Low handgrip strength	PR = 0.96	0.74–1.25	0.771
Medium IDDS tertile vs. Low	Relative muscle mass	β = 0.109	−0.184–0.401	0.466
High IDDS tertile vs. Low	Relative muscle mass	β = 0.365	0.054–0.676	0.022

Footnote: Models adjusted for age, sex, academic program, physical activity, and pre-existing health conditions. Robust variance estimators were applied.

Table S8. Adjusted associations between PCA-derived dietary patterns and excess body weight, low handgrip strength, and relative muscle mass.

Dietary pattern (reference: Low-Intake)	Outcome	Adjusted effect	95% CI	p-value
Energy-Dense/Ultra-Processed	Excess body weight	PR = 1.01	0.74–1.38	0.954
Prudent/Whole-Food	Excess body weight	PR = 1.26	0.88–1.80	0.207
Energy-Dense/Ultra-Processed	Low handgrip strength	PR = 0.89	0.63–1.25	0.495
Prudent/Whole-Food	Low handgrip strength	PR = 1.11	0.70–1.78	0.651
Energy-Dense/Ultra-Processed	Relative muscle mass	β = 0.094	−0.226–0.413	0.566
Prudent/Whole-Food	Relative muscle mass	β = −0.297	−0.826–0.233	0.273

Footnote: Fully adjusted models showed no independent associations between PCA-derived dietary patterns and study outcomes.
